# Obesity contributes to hepatocellular carcinoma development via immunosuppressive microenvironment remodeling

**DOI:** 10.3389/fimmu.2023.1166440

**Published:** 2023-05-17

**Authors:** Jian Yang, Jialuo He, Yiting Feng, Ming Xiang

**Affiliations:** Department of Pharmacology, School of Pharmacy, Tongji Medical College, Huazhong University of Science and Technology, Wuhan, China

**Keywords:** obesity, tumor immune microenvironment, immune dysfunction, metabolic shift, fatty acid, immunotherapy

## Abstract

It is generally recognized that the initiation of obesity-related hepatocellular carcinoma (HCC) is closely associated with hepatic inflammation. However, the paradoxical role of inflammation in the initiation and progression of HCC is highlighted by the fact that the inflammatory HCC is accompanied by significant immune effector cells infiltration compared to non-inflammatory HCC and HCC with enhanced immune response exhibits better survival. Importantly, the cancer progression has been primarily attributed to the immunosuppression, which can also be induced by obesity. Furthermore, the increased risk of viral infection and thus viral-HCC in obese individuals supports the view that obesity contributes to HCC via immunosuppression. Here, we have reviewed the various mechanisms responsible for obesity-induced tumor immune microenvironment and immunosuppression in obesity-related HCC. We highlight that the obesity-induced immunosuppression originates from lipid disorder as well as metabolic reprogramming and propose potential therapeutic strategy for HCC based on the current success of immunotherapy.

## 1 Introduction

Obesity, usually caused by an imbalance between energy intake and expenditure, was defined as an epidemic by WHO in 1997 and is widely prevalent both in developed and developing countries (1). Based on an abnormal metabolic environment, obesity results in altered immune functions, initially in the form of low-grade chronic systemic inflammation, and can develop into immune dysfunction (1, 2). Obesity is an independent risk factor for many types of cancer, and predominantly in liver and pancreatic cancer (3). Liver cancer is the fifth most commonly occurring cancer in the world and the third leading cause of mortality, and hepatocellular carcinoma (HCC) accounts for 80% of liver cancers (4). In a cohort study including 900,000 US people, male with a BMI ≥35 kg/m^2^ had 4.5 times the lethal risk of liver cancer than those with a normal BMI (≤25 kg/m^2^, 3). In addition, findings of another study, which consisted of 7 million participants indicated that even every 5 kg/m^2^ increase in BMI upregulated the risk rate of liver cancer by 24%, underscoring the potential association between obesity and HCC development (5).

Generally speaking, obesity contributes to the initiation of HCC in three ways. 1) Excessive fat deposition in adipose tissue can lead to upregulation of pro-inflammatory adipokines and downregulation of anti-inflammatory adipokines, thereby resulting in chronic inflammation. Chronic and mild inflammation can induce local or systemic insulin resistance (IR), which in turn induces high levels of insulin and IGF-1, thus stimulating abnormal hepatocyte proliferation (6, 7). 2) Chronic inflammation and hepatic lipid infiltration can cause cellular damage and oxidative stress in hepatocytes through multiple mechanisms, and chronic oxidative stress promotes DNA damage and compensatory repair processes, thus facilitating gene mutation and oncogenesis (8). 3) Obesity is directly related to the occurrence of non-alcohol fatty liver disease (NAFLD), which will progress into non-alcohol steatohepatitis (NASH), hepatic fibrosis or even hepatic cirrhosis if the symptom is not relieved (9). Hence, chronic liver injury leads to a cycle of cell death-repair-fibrosis, in which pre-HCC cells undergo malignant transformation and lead to tumorigenesis (10). These predisposing factors can occur both individually or simultaneously during obesity.

Inflammatory HCC, usually in the early stage, characterized by infiltration of CD8^+^ T cells as well as M1 macrophages, implies strong antitumor immunity, and HCC with enhanced immune response exhibits better survival (11). Conversely, non-inflammatory HCC, characterized by infiltration of Treg cells and M2 macrophages, is more prone to TP53 mutations and thus recruitment of immunosuppressive cells (11). Considering that the liver is rich in immune cell infiltration which usually indicates an effective antitumor effect, shifting from early inflammation to lately immunosuppressive environment in obesity-related HCC is critical for HCC progression (12). Since the function of immune cells largely depends on their metabolic pattern, which can be easily disrupted by metabolic dysfunction, revealing the possible impacts of obesity-induced lipid disorder on immune function is meaningful (13). Hence, this review focuses on the crucial role of obesity in HCC initiation, tumor immune microenvironment (TIME) formation, immunosuppression, and immunotherapy during HCC progression, and aims to provide potential immunotherapeutic targets and treatment strategies.

## 2 Obesity remodels TIME

TIME is essential for both HCC development and immunosuppression (14). The close proximity of the liver to visceral adipose tissue facilitates the transportation of the adipose metabolites to liver via the portal vein and lymphatic vessels, and thus the HCC TIME can be easily influenced by adipose tissue (15, 16). As the neovascular system is unable to keep pace with the rapid adipose expansion and results in reduced adipose tissue blood flow, adipocytes can rapidly reach the limit of oxygen diffusion, triggering adipose tissue hypoxia, which is an early determinant of adipose tissue dysfunction and may lay foundation for local hypoxia in liver via disturbing hepatic oxygen gradients (17–19). Correspondingly, high-fat diet increases hypoxia-inducible factor 1α (HIF-1α) and vascular endothelial growth factor (VEGF) expression, thus leading to increased lactate production and lactate accumulation in adipocytes (20). Excessive fat accumulation inhibits lactate dehydrogenase β (LDHβ), thereby blocking the conversion of lactate to pyruvate and contributing to lactate accumulation in adipose tissue (21). Adipocyte lactate production is often accompanied by glucose consumption, which is further promoted under conditions of obesity-induced increase in HCC glycolysis. Although obesity usually implies excessive calorie intake, carbohydrates tend to be taken up by adipocytes and HCC cells for glycolysis, leading to nutrient limitation in TIME (22–24). As a result, there is a lack of sufficient nutrients in TIME to support rapid proliferation of immune cells for antitumor immunity. Glucose deprivation can also induce T cell exhaustion and immune escape (25). For instance, low glucose environment facilitates the conversion of effector T cells to Treg cells through inducing forkhead box P3 (FOXP3) expression and activation (26). The function of NK and dendritic cells is markedly impaired by low glucose, and reduced HIF-1α-mediated secretion of pro-inflammatory cytokines is responsible for it (27). In addition, lactate inhibits T cell functions by suppressing T cell proliferation as well as interferon γ (IFN-γ) production (28). Moreover, lactate uptake is not necessary for peripheral Tregs but found to be required for intratumor Tregs for its role in metabolic support (29). A recent study has shown that lactate in high glycolytic environment upregulated programmed cell death protein 1 (PD-1) levels in Tregs, and anti-PD-1 therapy reactivates Tregs, thus leading to therapy failure (30). In addition, lactate drives M2 polarization in TIME via the mitochondrial pyruvate metabolism (31).

Thus, obesity involves in remodeling immunosuppressive tumor microenvironment by modulating adipose tissue metabolism. Under these conditions, liver-infiltrating immune effector cells exhibit a restricted proliferative capacity and tend to be immunosuppressive due to metabolic adaptations.

## 3 Obesity interrupts adipokine balance and facilitates HCC initiation

Adipokines are a group of cytokines secreted by the adipose tissue, and exhibit pleiotropic effects on the metabolism, signal transduction and inflammatory pathways. HCC initiation could be primarily attributed to the adipokine imbalance, which is directly related to the systemic inflammation, metabolic disorder and immune dysfunction ( **Table 1**).

**Table 1:** Mechanisms responsible for obesity-induced adipokines dysfunction and their roles in obesity-related HCC.

Adipokines	Functions	Tendency in obesity	Inducers	Effects on HCC	Molecular mechanisms	References
Anti-inflammatory						
Adiponectin	Increases insulin sensitivity Relieves inflammation Eliminates HCC cells	Downregulated	Insulin resistance Hypoxia microenvironment Chronic inflammation	Inhibits	AMPK/TSC2/mTOR↑, Caspase-3↑, JNK↑, STAT3↓, PI3K/Akt↓, SOCS3↑, TNF-α↓.	(20–22, 24–29)
Ghrelin	Regulates energy steady state Maintains healthy liver function Reduces lipid toxicity	Downregulated	Insulin resistance Positive energy balance	Inhibits	NF-ĸB↓, TNF-α↓, TG↓.	(30–33)
Irisin	Improves glucose homeostasis Improves insulin resistance Induces weight loss	Downregulated	Lack of exercise T2D	Inhibits	*De novo* lipogenesis↓	(31, 34, 35)
Pro-inflammatory						
Leptin	Regulates the appetite and energy balance Resists weight gain Promotes hepatic steatosis	Upregulated	Lipotrophy Chronic inflammation	Promotes	JAK2/STAT3↑, PI3K/Akt↑, ERK↑, p53/FOXO3A↑	(24, 28, 36, 37)
Resistin	Antagonizes insulin Induces hepatic insulin resistance	Upregulated	Insulin resistance Central/visceral obesity	Promotes	p38 MAPK/NF-κB↑	(38–41)
Visfatin	Exerts insulin-like hypoglycemic effect Promotes adipose expansion Hepatic inflammation	Upregulated	Insulin resistance	Promotes	NF-ĸB↑, STAT3↑, PI3K/Akt↑, ERK↑	(31, 42–47)

### 3.1 Anti-inflammatory adipokines

#### 3.1.1 Adiponectin

Adiponectin is primarily produced in the adipose tissue of lean subjects, and its secretion is usually inversely related to BMI in obesity (32). Mechanistically, obesity induces IR and thus inhibits adiponectin secretion through PI3K/FoxO1 pathway (33). In addition, lipid deposition in adipose tissue induces a hypoxic microenvironment, which inhibits adiponectin transcription through HIF-1α pathway (34). Chronic inflammation caused by obesity leads to increased secretion of tumor necrosis factor-α (TNF-α), IL-6, IL-18, and other Th1 cytokines that can also inhibit adiponectin (35). As an anti-inflammatory adipokine, adiponectin levels are positively related to Th2 cytokines such as IL-10 (48). Adiponectin suppresses the HCC progression *in vivo* by inhibiting cell proliferation and inducing cell apoptosis, and antagonizes carcinogenic effects of leptin (36). In addition, adiponectin activates tuberous sclerosis complex 2 (TSC2) protein through receptor-mediated phosphorylation of 5’- adenosine monophosphate-activated protein kinase (AMPK), thus attenuating the phosphorylation of mammalian target of rapamycin (mTOR), and directly protecting against HCC (37, 49). Adiponectin also eliminates HCC cells by activating caspase-3 and increasing the phosphorylation of c-Jun N-terminal kinase (JNK) (50). Moreover, adiponectin regulates ceramide metabolism, promotes insulin sensitivity, reduces inflammation, supports hepatocyte survival and inhibits tumor growth (51). Adiponectin attenuates leptin-induced signal transducer and activator of transcription 3 (STAT3) and protein kinase B (Akt) activation by upregulating suppressor of cytokine signaling 3 (SOCS3), a physiological negative regulator of leptin signal transduction, thus inhibiting leptin-induced hepatoma cell proliferation (38). However, low adiponectin levels restrict inhibitory effect of TNF-α on tumor cell proliferation by inhibiting the production of TNF-α in macrophages (49). Above all, adiponectin plays a vital role in maintaining homeostasis and its downregulation predicts elevated risk of obesity-related HCC.

#### 3.1.2 Ghrelin

Ghrelin is upregulated in under-nourished states, such as anorexia nervosa, and is downregulated in conditions linked with positive energy balance, such as obesity (39). Obesity-induced IR is associated with decreased ghrelin levels (40). Sufficient serum ghrelin levels play vital roles in maintaining healthy liver function. Ghrelin induces immunosuppression via facilitating M2 and Treg phenotypes (41). Thus, ghrelin decreases the levels of Th1 cytokines including TNF-α, IFN-γ, IL-1β, IL-6 and increases Th2 cytokines including IL-10, IL-4, transforming growth factor (TGF-β), thereby inhibiting hepatic inflammation and HCC initiation (41). In mouse models, ghrelin inhibits HCC progression by reducing TG content and cytokines such as TNF-α as well as IL-6, and alleviates lipotoxicity by stimulating autophagy and inhibiting nuclear factor-κB (NF-ĸB) pathway (40). Notably, circulating ghrelin levels in obese people were found not to be reduced by a meal (52).

#### 3.1.3 Irisin

Irisin was initially regarded as a hormone secreted by skeletal muscles in response to the tremor and movement stimuli, and further studies revealed that irisin was also present in other healthy tissues (42). It plays a critical role in the metabolism regulation, improves glucose homeostasis, IR and induces weight loss (40). Thus, irisin exhibits a positive effect on obesity, hyperlipidemia and hyperglycemia caused by the metabolism dysfunction. In addition, irisin acts as an important negative regulator of cancer and inhibits the proliferation, migration and invasion of cancer cells (43). In HCC cells, fibronectin type III domain-containing protein 5 (FNDC5), a precursor of irisin, was found to regulate gene expressions involved in lipogenesis, tumorigenesis as well as inflammation, and increased irisin levels might restrict HCC development via inhibiting *de novo* lipogenesis (42). Importantly, irisin levels have been shown to decrease in more advanced HCC (44). Moreover, circulating irisin levels have been observed to increase in individuals who engage in exercise-inducing activities, but they gradually decrease in those who are sedentary and lack exercise, suggesting that exercise could be a promising prevention for obesity-related HCC (45). However, irisin secretion is significantly hampered in obesity and Type 2 diabetes mellitus (T2D), explaining underlying cause for increased risk of obesity-related HCC (53).

### 3.2 Pro-inflammatory adipocytokines

#### 3.2.1 Leptin

Leptin is a multifunctional adipokine that regulates appetite and energy balance (46). Although leptin is supposed to resist the weight gain, obesity usually leads to increased secretion of leptin (47). Inflammatory activation increases leptin synthesis and release (54). In turn, leptin contributes to IR, hepatic steatosis, and fibrosis, thus playing a vital role in regulating immune responses, glucose homeostasis, and angiogenesis (55). Studies have reported that leptin is a key regulator of HCC development and ectopic serum leptin levels are regarded as a hallmark of metabolic disorders leading to HCC (36). Leptin promotes the proliferation and inhibits the apoptosis of hepatoma cells (51). Moreover, it can enhance mitosis, invasion, and metastatic potential of HCC cells by activating the JAK2/STAT3, PI3K/Akt, and ERK pathways (36). Besides, leptin induces autophagy through affecting the p53/FOXO3A axis, thereby eliminating apoptosis (56). Interestingly, studies have also shown that leptin-leptin receptor (OBR) is involved in the angiogenesis of HCC, thus facilitating the progression of NASH to HCC (57). Moreover, leptin acts on monocytes/macrophages by inducing the synthesis of eicosanoid, NO, and several Th1 cytokines (58). In addition, leptin induces neutrophil chemotaxis and stimulates the release of oxygen free radicals as part of the immune response and host defense mechanisms. A recent study reported that inhibition of ATX-LPA-Lpar2-p38-leptin axis in the mouse HCC model can inhibit tumor growth (59). Thus, obesity, characterized by hyperleptinemia and central leptin resistance, directly increases the risk of HCC.

#### 3.2.2 Resistin

Resistin is a pro-inflammatory adipokine mediating hepatic insulin resistance, and named for its role in antagonizing insulin (60). Resistin has been associated with the progression, angiogenesis, chemical resistance and increased risk of metastasis in various cancer models (61). Activation of p38 MAPK/NF-κB signaling pathway is responsible for resistin-mediated declined HCC cell adhesion and thus metastasis (62). In addition, meta-analysis results have suggested that high resistin levels are related to an increased risk of obesity-related cancer (63). Since serum resistin levels were positively correlated to the central/visceral obesity (but not BMI) and IR, resistin may be involved in driving obesity-related HCC, though further investigations are needed (64).

#### 3.2.3 Visfatin

Visfatin is a cytokine mainly secreted by the visceral adipose tissue and functions both as an extracellular factor and an intracellular enzyme (65). It exerts insulin-like hypoglycemic effect through binding to the insulin receptor and promotes adipose tissue differentiation and proliferation (66). Visfatin is upregulated by cytokines which promote IR such as TNF-α and IL-6, and thus elevated in T2D and IR (40, 67). Increased visfatin levels in turn induce IR and hepatic inflammation via NF-ĸB and STAT3 pathway (68). Interestingly, visfatin levels were also associated with the severity of hepatic steatosis and fibrosis, thus facilitating HCC progression. It has been reported that visfatin increases miR-21 expression to promote HCC migration (69). Moreover, visfatin also plays a crucial role in hepatocyte proliferation (70). Recent studies have also shown that enhanced invasion in liver cancer cells caused by visfatin could be attributed to PI3K/Akt and ERK signaling cascades (71). Hence, obesity results in upregulated serum visfatin levels and thus potentially increases the risk of HCC (72).

## 4 Obesity induces dysfunction of immune effector cells

Immune effector cells engaged in anti-cancer immunity primarily include CD4/8^+^ T cells, macrophages, B cells and nature killer (NK) cells. However, these cells undergo function loss and numeric decrease owning to the metabolic dysfunction and lipotoxicity (**Table 2**).

**Table 2:** Mechanisms responsible for altered functions of immune cells in obesity-related HCC.

Cells	Tendency in obesity-related HCC	Mechanisms	References
Immune Effector Cells			
CD8^+^ T cells	Suppressed	Metabolic shift from OXPHOS to glycolysis reduces reliance of CD8^+^ T cells on oxygen but makes them become more sensitive to glucose deprivation in TIME. Obesity also impairs T cell compartment and induces T cells exhaustion.	(73–78)
CD4^+^ T cells	Suppressed	Although elevated circulating CD4^+^ T cells are induced by leptin, CD4^+^ T cells display increased expression of exhaustion markers. CD4^+^ T cells possess greater mitochondrial mass when compared to CD8^+^ T cells and could be easily impaired by lipid-induced ROS burst.	(78–82)
B cells	Suppressed	B cell activation facilitates NAFLD/NASH progression and thus HCC. During obesity, adipocytes promote MDSCs development and inhibit B lymphopoiesis. Persistent inflammation hampers response of B cells to antigens and accelerate age defects in B cells. Increased lipolysis facilitates Bregs survival and thus immunosuppression.	(83–92)
NK cells	Paradoxical	Lipid deposition and aberrant lipid metabolism lead to senescence of iNKT in the TIME. Metabolic shift based on lipid-rich environment further hampers functions of NK cells. However, cholesterol accumulation in NK cells may also stimulates effector functions and thus inhibits HCC progression.	(93–96)
Immunosuppressive Cells			
MDSCs	Paradoxical	MDSCs undergo metabolic shift from aerobic glycolysis to FAO in lipid-rich environment to increase the utilization of FA, thus meeting high energy demand and sustaining suppressive function in TIME. Cholesterol accumulation also enhances functions of MDSCs, but its derivatives block MDSCs survival and abundance.	(97–100)
TAMs	Enhanced	In glucose-rich environment such as hyperglycemia, TAMs possess upregulated GLUT1 expression, thus enhancing their glycolysis and immunosuppressive functions. In low glucose but lipid-rich environment such as obesity-related TME, TAMs take full advantage of fatty acids to maintain immunosuppressive functions.	(101–106)
Tregs	Paradoxical	Although obese populations exhibit diminished frequency of Tregs, intratumoral Tregs can be distinct with peripheral Tregs. In lipid-rich environment, intratumoral Tregs upregulate their surface FA transporter CD36 and show a tendency towards FAO, thus meeting biomass demands and exhibiting resistance to lipotoxicity.	(107, 108)

### 4.1 CD8^+^ T cells

CD8^+^ T cells are the major component against tumor progression among anti-tumor immunity system. Once activated, the increased requirement for biomass and energy results in metabolic shift from oxidative phosphorylation (OXPHOS) to aerobic glycolysis, and thus robust proliferation (73). Since obesity is usually accompanied by IR and thus increased levels of blood glucose, this fills the need for glucose and thus facilitates glycolysis in CD8^+^ T cells (74). Moreover, increased leptin levels also promote glycolysis in CD8^+^ T cells via PI3K/Akt/mTOR pathway, which partly accounts for obesity-induced chronic inflammation (75). The metabolic shift reduces reliance of CD8^+^ T cells on oxygen for energy acquisition and thus renders them to retain their immunological function even after migrating into environments with poor oxygen, such as hypoxic TIME within HCC. Importantly, the reliance on glycolysis causes CD8^+^ T cells become more sensitive to glucose deprivation, which leads to decreased production of IFN-γ, granzyme B and perforin in CD8^+^ T cells (109). Moreover, increased leptin levels and availability to fatty acid (FA) also results in robust STAT3 signaling, which in turn promotes fatty acid β oxidation (FAO) and inhibits glycolysis due to glucose deprivation in TIME, leading to disability of CD8^+^ T cells to restrict tumor proliferation (110). Hence, obesity remodels TIME and promotes migration of CD8^+^ T cells into TIME partly via metabolic shift, thus leading to suppressed immunity. Many studies have indicated that obesity induces harmful effects on T cell compartment, including decreased frequency of T cell progenitors in the thymus, thymic involution as well as restricted TCR diversity, and results in increased infiltration of PD-1^+^ exhausted CD8^+^ T cells in adipose tissue and liver (76–78). In mice with HFD, CD8^+^ T cells exhibited downregulated expression of Ki-67, inducible co-stimulator (ICOS) and granzyme B when compared to mice with chow diet, and altered FA partitioning could be responsible for the reduced function of CD8^+^ T cells (111). The function of CD8^+^ T cells could be also impaired by deposition of unsaturated fatty acids (such as cholesterol) via lipid peroxidation (112). Moreover, a recent study has also reported that caloric restriction delayed immune senescence and increased levels of hepatic CD4^+^ and CD8^+^ T cells in obesity-related HCC via shaping gut microbiome (113).

### 4.2 CD4^+^ T cells

Many prior studies have reported that CD4^+^ T cells inhibit HCC initiation and restrain tumor progression (79, 114). Obesity usually increases circulating CD4^+^ T cell levels via leptin-mediated upregulation of major histocompatibility complexes-II (MHC-II, 80). However, CD4^+^ T cells derived from obese mice displayed inhibited proliferation and cytokine production when stimulated *ex vivo*, and same results were obtained in humans (78). This may be attributed to increased expression of exhaustion markers such as PD-1, LAG-3 and Tim-3. CD4^+^ T cells possess greater mitochondrial mass compared to CD8^+^ T cells and thus produce more mitochondria-derived ROS, which meant that CD4^+^ T cells could be easily impaired by mitochondrial dysfunction (79, 81). Hence, increased levels of FA especially linoleic acid, can cause mitochondrial oxidative stress and mediate selective loss of CD4^+^ T cells in the liver, thus inducing liver carcinogenesis (81). Besides, increased availability to FA also activates peroxisome proliferator-activated receptor-α (PPAR-α) pathway and leads to carnitine palmitoyltransferase upregulation, which in turn facilitates influx of FA into mitochondria and leads to greater apoptosis of CD4^+^ T cells via ROS burst (82). Moreover, reduced tumor infiltration of CD4^+^ T cells caused by ROS burst might also impair immunotherapy targeting liver tumors, suggesting that overcoming FA-induced impairment could be a potential strategy for obesity-related HCC (115).

### 4.3 B cells

During obesity, activation of B cells is directly related to systemic inflammation via antigen presentation and pro-inflammatory cytokine secretion (116). Increasing evidence indicates that intrahepatic B cells play key roles in NAFLD progression (83). In several experimental models and patients, NASH is characterized by B cell infiltration, suggesting that obesity may facilitate B cell activation and thus exacerbate NAFLD or NASH, which is important risk factor for obesity-related HCC (84, 85). However, obesity also impairs B cell biology. Mice with HFD exhibit decreased frequency of B cells in bone marrow (86). Mechanistically, adipocytes secrete soluble factors and facilitate MDSCs development, thereby inhibiting B lymphopoiesis (117). Although B cells from obese subjects secrete more pro-inflammatory cytokines, they also exhibit higher senescence-associated secretory phenotype (SASP) markers (87, 88). Moreover, the persistent high inflammation in obesity restrains proper regulation of B cell responses to novel antigens, such as cancer cell markers (89). Increased inflammation and persistent immune activation induced by obesity also accelerate age defects in B cells (88). Importantly, infiltration of B cells positively correlates with HCC progression in chronic liver injury, and B cells from mice fed with HFD disrupted antitumor immunity in NASH-driven HCC (84, 90). The possible reason is that fatty acids caused by enhanced lipolysis promote survival of regulatory B cells (Bregs), which could accelerate HCC progression (91, 92). However, it doesn’t explain the reduction of Bregs in obesity, and further studies about the roles of B cells in obesity-related HCC are needed (118).

### 4.4 NK cells

Circulating NK cells in obese populations display increased CD69 expression, which indicates chronic activation, ultimately leading to diminished NK cell cytotoxic activity (119). Liver infiltrated-NK cells during obesity also tend to possess less cytotoxic ILC-1 phenotype and may explain increased risk of HCC in obese populations (120). Dominik Pfister et al. indicated that thirty percent of mice with choline-deficient high-fat diet developed HCC with similar genetic alterations of human NAFLD-HCC, but anti-PD-1 therapy failed to regress tumor burden in spite of increased infiltration of immune effector cells, and this effect could be attributed to impaired function of CD8^+^ and NK cells (121). Lipidomic profiling revealed the accumulation of long-chain acylcarnitines (LCACs) and aberrant lipid metabolism in HCC tissues, which finally led to senescence of invariant natural killer T cells (iNKT) in TIME (93). Similarly, lipid accumulation in NK cells cause metabolic shift based on lipid-rich environment in obesity, thus hampering NK cell functions (94). Increased uptake of FA and cholesterol might be also responsible for impairment of NK cells in HCC and blocking transport of lipids to mitochondria could effectively restore NK cell cytotoxicity and restrain tumor growth (95). However, the role of cholesterol could be controversial since a prior study has also reported that cholesterol accumulation in NK cells stimulates effector functions and thus inhibits HCC progression (96).

## 5 Obesity enhances function of immunosuppressive cells

In contrast of immune effector cells, immunosuppressive cells including myeloid-derived suppressive cells (MDSCs), tumor-associated macrophages (TAMs) and regulatory T cells (Tregs) are able to adjust to lipid-rich environment as well as TIME, and thereby can facilitate immunosuppression. FAO plays a vital role in this process.

### 5.1 MDSCs

MDSCs are a highly heterogeneous group of immature myeloid immune cells, which include the naïve macrophages, granulocytes and dendritic cells (122). Inflammatory-cell cycle-related kinase (CCRK) circuitry under obese circumstance drives immunosuppressive metabolic reprogramming and enhances polymorphonuclear MDSCs recruitment as well as tumorigenicity, thereby facilitating obesity-related HCC progression (123). Although lipid deposition leads to metabolic dysfunction in immune effector cells and thus immunosuppression, FA availability in TIME enhances the function of MDSC. Enhanced FAO facilitates infiltration of MDSCs via secreting complement C3, whereas suppression of FAO results in MDSCs inhibition (124). Moreover, in lipid-rich environment, MDSCs can reprogram their metabolism and transfer from aerobic glycolysis to FAO to reduce their reliance on glucose and thereby increase the utilization of FA, thus meeting high energy demand and sustaining suppressive function in TIME, whereas genetic depletion of CD36 can hamper lipid uptake and inhibit MDSCs (97–99). Obesity promotes hepatic inositol requiring enzyme 1 α (IRE1α) activation and X-box binding protein 1 (XBP1)-drived production of cholesterol, which in turn elicits immunosuppression by enhancing the functions of MDSCs (100). In addition, obesity elevates CXCL1 levels in TIME and promotes the infiltration of MDSCs in tumor sites, thereby inducing cell death of CD8^+^ T cells (125). However, increased cholesterol accumulation under oxidative stress status also stimulates the generation of the different cholesterol derivatives. These cholesterol derivatives, especially oxysterols, could activate liver X receptor (LXR)-ApoE axis and block MDSCs survival and abundance (126). Hence, caution is needed if MDSCs metabolism is targeted for therapeutic purposes.

### 5.2 TAMs

Generally, TAMs are mixed intratumoral macrophage populations with different phenotypes and are generally categorized into M1/M2 macrophages, though more precise classification is needed (127). Many studies have indicated that glucose transporter 1 (GLUT1) is upregulated in TAMs and leads to increased glycolysis (101, 102). It is not surprising that hyperglycemia in obesity facilitates glycolysis in TAMs and results in immunosuppression via inducing chemokine and programmed cell death protein ligand 1 (PD-L1) expression (103). However, glucose may be also scarce in TIME as highlighted above, suggesting that glycolysis in TAMs may be restrained. Under this condition, obesity-induced M2-like TAMs are largely dependent on increased availability to FA. Lipid droplets originated from FA are essential organelles in TAMs for FA metabolism and sustain immunosuppressive phenotype (104). FAO is critical for maintaining the function of TAMs since its inhibition can impair IL-4-induced M2 activation (105). Enhanced FAO can also promote mitochondrial OXPHOS and ROS production, leading to STAT6 activation and transactivation of genes related to TAM generation and function (106). However, a recent study based on HCC model indicated that decreased FAO caused by fatty acid binding protein 5 (FABP5) leads to accumulation of lipids in macrophages and fosters immune tolerance, suggesting that FABP5 may act as a potential biomarker of TAMs (128). Importantly, increased lipid deposition also facilitates ROS production via lipid peroxidation, thus in turn promoting FAO via ROS-Caspase1-PPAR pathway and leading to liver carcinogenesis (129). Hence, declined FAO may potentially serve as a bad marker of M1 macrophages since it provides signaling molecules and sufficient lipids for TAM initiation. A recent study highlighted the vital role of GSK3β in TAMs, since GSK3β deficiency in macrophage can restrict HCC progression by inhibiting M2 phenotype and enhancing the sensitivity of anti-PD-1 immunotherapy (130). Overall, considering that obesity is usually accompanied by increased serum insulin levels, which are known to activate GSK3β, there is still a possibility that obesity can facilitate M2-like phenotype and thereby promote HCC progression in certain circumstances.

### 5.3 Tregs

Tregs are a common obstacle against anti-tumor therapies. While Tregs are usually enriched in lean visceral adipose tissue, obese populations exhibit diminished frequency of Tregs (131). However, intratumoral Tregs can be distinct with peripheral Tregs. Tregs show a tendency towards FAO and exhibit resistance to lipotoxicity (107). In the obese, intratumoral Tregs can upregulate their surface FA transporter CD36 to obtain sufficient biomass and adapt to the lipid-rich environment (108). Increased FA uptake can then induce PPAR-dependent lipid metabolism and thus enhance Tregs viability as well as function. Moreover, a recent study indicated that neutrophil extracellular traps are abundant in livers with steatohepatitis and promote Treg differentiation through metabolic reprogramming, thereby affecting the balance of Th17/Treg cells and contributing to liver carcinogenesis (132). FOXP3 reprograms T cell metabolism to enhance OXPHOS and inhibit glycolysis, thus rendering Tregs advantageous in obesity-induced TIME (133). Insulin signaling plays a dual role in regulating the functions of Tregs. For example, although insulin was found to drive Tregs in a HIF-1α–dependent manner, obesity-induced hyperinsulinemia can impair the ability of Tregs to secrete IL-10 (134, 135). However, increased levels of IL-6, IL-1β and TGF-β can impair T cells proliferation and induce their polarization to Tregs.

## 6 Obesity affects cytokine secretion to facilitate HCC progression

Cytokines are critical for obesity-related pathology. However, most of them play dual roles in regulating inflammation and HCC. We here introduce four distinct cytokines including TNF-α, interleukins, TGF-β and chemokines. All of them are secreted abnormally in obesity due to dysfunction of adipose and immune cells, thus facilitating inflammation in early stage but also promoting HCC progression via inducing immunosuppression.

### 6.1 TNF-α

The elevated serum TNF-α levels are generally attributed to increased secretion by macrophages as well as immune cells (136). In obesity, excessive lipid deposition in adipocytes leads to increased cell apoptosis and causes increased accumulation of macrophages around dying adipocytes, regarded as crown-like structure, thus secreting TNF-α (137). TNF-α activates JNK pathway to impair insulin signaling and interacts with NF-κB to upregulate genes involved in regulating apoptosis, proliferation, inflammation, angiogenesis. Hence, increased TNF-α secretion is associated with inflammation and increased risk of HCC (137). Although increased inflammatory environment may cause damage to HCC cells, leptin has been shown to counteract toxicity exerted by TNF-α (138). Importantly, TNF-α can also promote lipolysis in adipocytes and facilitate release of FA, which plays a key role in development of lipid disorder and obesity-induced immunosuppression (139). However, TNF-α levels may be decreased in TIME due to impaired function of CD8^+^ T cells and macrophages.

### 6.2 Interleukins

Interleukins associated with tumorigenesis belong to an extremely large family which is still increasing as the number of papers exploring the roles of new members are being published. Among them, IL-6 and IL-1β are crucial for obesity-related HCC. Like TNF-α, IL-6 is related to the progression from steatosis to HCC, and its increased levels in obesity is related to several ways (140, 141). IL-6 facilitates cell proliferation and differentiation via promoting STAT3 activation, which is crucial for HCC development (142). Moreover, a recent study has reported that adipocyte-derived IL-6 sensitized macrophages to IL-4 signaling and thus facilitated M2 phenotype (143). Besides, IL-6 can also increase infiltration of Tregs in TIME and thus facilitate HCC progression, which could be suppressed by cystathionine β-synthase-mediated STAT3 inhibition (144). IL-1β is also associated with increased HCC risk and poor prognosis (145). Increased IL-1β levels are attributed to NF-κB activation and NOD-like receptor family, pyrin domain containing 3 (NLRP3) activity, which in turn could be stimulated in obesity by several factor such as FA, cholesterol, ROS and hyperglycemia (146). Although known as inflammatory factor, IL-1β can also lead to immunosuppression and IL-1β deficiency results in tumor regression (147). Blocking IL-1β enhanced effector immune cells and inhibited immunosuppressive cells, thus facilitating checkpoint inhibition therapy.

### 6.3 TGF-β

Increased TGF-β secretion can originate from high number of adipose-derived stem cells, which has been associated with adipose tissue expansion (148, 149). In obesity, TGF-β is an underlying contributor to IR via inducing cell hypertrophy and reducing the functions of islet β cells (150, 151). Besides, a recent study has also highlighted that TGF-β signaling in hepatocyte can inhibit white adipose tissue browning and thus facilitate obesity and NAFLD (152). In HCC cells, increased TGF-β levels induce epithelial-mesenchymal transition and reprogram lipid metabolism, thus promoting adaption of HCC to lipid-rich environment (153). In addition, TGF-β signaling works as a key regulator in immune cell differentiation, proliferation as well as survival and thus contributes to NAFLD and NAFLD-HCC (154). TGF-β can also induce immunosuppression through distinct mechanisms. For instance, in CD8^+^ T cells, enhanced TGF-β signaling can suppress IFN-γ expression to inhibit its cytotoxicity effects (155). TGF-β can suppress NK cells and thus inhibit the recruitment of immune effector cells to tumor. In addition, TGF-β drives M2 phenotype and Treg phenotype, leading to immunosuppression in HCC (156). Acid metabolites like lactate in TIME could also enhance TGF-β and its downstream signaling in Tregs to promote tumorigenesis (157). Importantly, TGF-β promotes PD-1 expression in antigen-specific T cells via SMAD3 activation, suggesting that increased levels of TGF-β in obesity could be a potential biomarker for anti-PD-1 therapy (158).

### 6.4 Chemokines

Chemokines can induce leukocyte chemotaxis and thus affect tumor behavior via immune regulation. CCL2 is mainly secreted by preadipocytes and adipocytes during obesity (159, 160). Increased CCL2 levels can lead to macrophage differentiation and accumulation in adipose tissue, thus promoting inflammation and inducing IR as well as hepatic steatosis. However, elevated CCL2 expression has been linked to loss of Kupffer cells and can cause increase of immature macrophages in liver, thus contributing to immunosuppressive microenvironment in liver cancer (161). Similarly, a recent study has also shown that CCL2 activation could also promote TAMs recruitment in TIME and facilitate HCC progression (162). CXCL-8 (also known as IL-8), another chemokine known to increase with the development of hepatic steatosis, is also involved in immunosuppression via MDSCs and TAMs recruitment, and contributes to the formation of TIME (163). Moreover, CCL22 has been reported to be induced by IL-6 and TNF-α, thereby stimulating Treg chemotaxis and accumulation (164). A recent study also indicated that excess serum lipid levels downregulate CXCR3 expression which promotes transfer of T cells into the tumor sites, thereby resulting in decrease of T cell infiltration in tumors (165).

## 7 Obesity impacts immunotherapy for HCC

Systemic therapy such as sorafenib is used as first-line therapy for HCC. In recent years, several immunotherapy regimens including immune checkpoint inhibitors (ICIs) and adoptive cell therapy have shown strong anti-tumor effects for HCC. Despite of the prevalence of obesity-related HCC, there are few studies that have focused on the potential impacts of obesity on immunotherapy of HCC.

### 7.1 ICIs therapy

Obesity appears to play a positive role in immunotherapy (mainly including ICIs therapy and adoptive cell therapy) of various cancers. ICIs therapy (such as anti-PD-1 and anti-CTLA4 antibodies) works by targeting depressed immune effector cells and enhancing their function, resulting in robust immune activation. In patients with NSCLC, high BMI was found to be independently associated with a better prognosis after atezolizumab treatment (166). A multicenter study also indicated that overweight predicted a better prognosis in a variety of cancers after ICIs therapy (167). Anti-PD-L1 & anti-angiogenic immunotherapy (atezolizumab in combination with bevacizumab) is currently the first-line treatment for advanced HCC. A subgroup analysis of survival outcomes based on the trials evaluating the efficacy of ICIs as first-line therapy showed that viral-HCC could benefit from ICIs (168). However, for non-viral-HCC patients, ICIs were not observed to be superior to sorafenib, which suggested that obesity may be not a positive marker for ICI therapy in HCC. However, all current clinical trials were not able to distinguish the subgroups of nonviral-HCC, thus implying that they also included cases of HCC related to NASH, obesity, alcohol-related, autoimmune hepatitis etc. Hence, the detailed classification of clinical cases of nonviral-HCC is necessary. In fact, a recent case with obesity and T2D showed that the efficacy of the regimen of atezolizumab combined with bevacizumab remained guaranteed and could overcome nivolumab tolerance (169). Notably, high BMI was related to the better prognosis upon anti-PD-1 therapy (including nivolumab, pembrolizumab, sintilimab, and toripalimab, 170). Patients in advanced HCC with BMI <25 had a worse median OS when treated with anti-PD-1 therapy in comparison to those with BMI ≥25 (171). This may be because, compared to healthy individuals, obesity predicts higher T-cell PD-1 expression, which may be associated with upregulated leptin (78). Thus, while obesity-induced T-cell PD-1 upregulation may predict systemic immunosuppression, this might imply greater sensitivity to anti-PD-1 therapy. However, a recent study highlighted that GSK3β activation in macrophage restricted anti-PD-1 immunotherapy in HCC (130). Since obesity is usually accompanied by hyperinsulinemia, obesity could also serve as a poor marker for anti-PD1 immunotherapy. Of note, obesity appears to increase the risk of side effects after ICIs therapy. Since ICIs enhance body’s immune system, they can often lead to autoimmune reactions. A recent study, which included 13,480 cases showed that obesity predicted a higher risk of colitis (172). Another study also revealed that obesity was related to an increased risk of immune-related adverse effects (irAEs) after nivolumab treatment (173). For patients treated with pembrolizumab, elevated BMI was associated with an increased risk of irAEs (174). However, irAEs can also predict a stronger immune response. In another study, Jacobo Rogado et al. reported that in patients with advanced cancer treated with single-agent anti-PD-1 antibody nivolumab or pembrolizumab immunotherapy, overweight was associated with greater outcomes, and the observed treatment benefit was significantly enhanced when irAEs were present in the overweight population (175). Importantly, due to the limitation of BMI in descripting the detailed information related to obesity, people should be more concerned about the impact of body composition on immunotherapy. For example, people with myosteatosis caused by excessive lipid accumulation in the muscle are associated with higher risk of failure after immunotherapy as well as a poor prognosis in HCC (176). Likewise, myosteatosis can predict strong toxicity of nivolumab.

### 7.2 Adoptive cell therapy

Adoptive cell therapy works by modifying immune effector cells (such as T cells) to express specific immune effector marker (such as chimeric antigen receptor), resulting in enhanced anti-tumor immunity in patients. Currently, studies about the effects of obesity on adoptive cell therapy for HCC are lacking. Wenshu Tang et al. observed that obesity-induced hypercholesterolemia can effectively impair NK cell function, whereas Wenhao Qin et al. indicated that high cholesterol increased NK cell lipid accumulation thereby enhancing their function (95, 96). Thus, the effect of obesity on allogeneic NK cell adoptive therapy may be positive or negative. However, there is a lack of sufficient data to determine the extent of cholesterol accumulation and the duration of cholesterol accumulation, which may account for the differential effects of cholesterol on NK cells. However, a recent study suggested that NK cell therapy could display potential efficacy in obesity-related cancer (177). Recently it has been found that GPC3-CAR-T cell therapy based on the GPC3 target of HCC cells was effective in clinical HCC treatment. Manuel Garcia-Jaramillo et al. reported that western diet increased hepatic GPC3 expression in the male mice (178). In addition, IR was also associated with increased levels of hepatic GPC3 expression, suggesting that obesity may predict better response to GPC3-CAR-T cell therapy (179).

## 8 Conclusion

**Figure 1:**
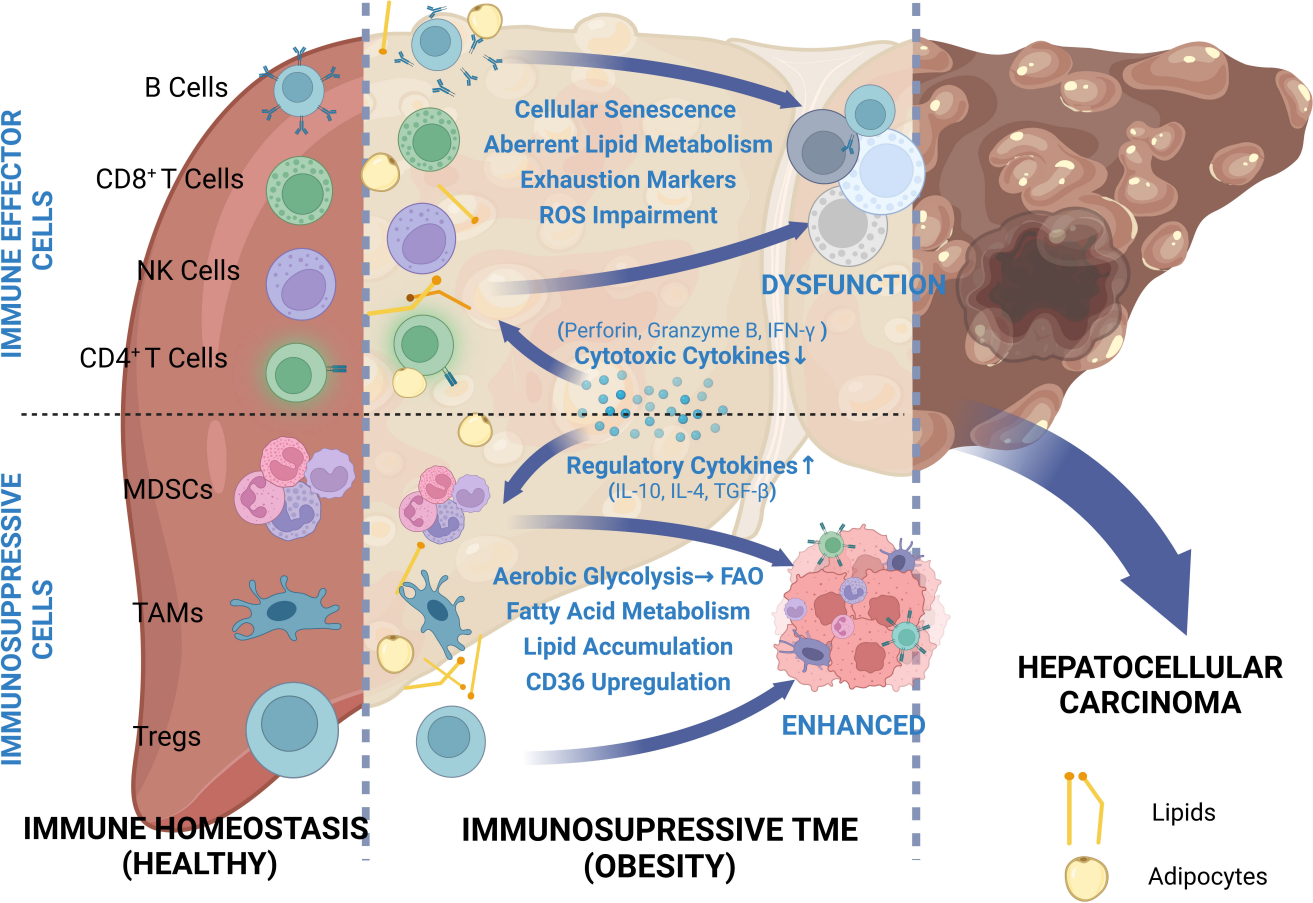
Comprehensive review of depressed cellular immunity in obesity-related HCC. Due to the proximity of the liver to visceral adipose tissue, the HCC TME can be easily influenced by adipose metabolites. In order to adapt to this abnormal metabolic environment, immune cells undergo metabolic shift and thus phenotypic change. While immune effector cells are depressed due to restricted glycolysis and exhausted marker expressions, immunosuppressive cells take full advantage of increased availability to lipids and exhibit enhanced functions.

Overall, based on the general understanding, obesity-related HCC is primarily attributed to NAFLD and IR. However, there are few studies that have focused on the inhibitory effects of obesity-induced metabolic disorder on antitumor immunity. In this review, we have presented a comprehensive overview about the distinct mechanisms through which obesity facilitates HCC progression via remodeling TIME and inducing immunosuppression (**Figure 1**).

Obesity-related immunosuppression is mechanistically related to the disrupted balance between adipocytes, hepatocytes, and immune cells. Excessive lipid deposition in visceral adipose tissue is the trigger for obesity-related HCC, and can directly lead to adipose tissue expansion (180). Thus, the body possess decreased anti-inflammatory adipokines levels and increased pro-inflammatory adipokines levels, thereby facilitating local or systemic inflammation, which is crucial for NAFLD-driven HCC (37). In this process, anti-inflammatory adipokines exert anti-tumor effects by inducing apoptosis or inhibiting expression of pro-tumor cytokines, whereas pro-inflammatory adipokines promote HCC progression via facilitating autophagy, angiogenesis and proliferation. Obesity-related inflammation and IR can also promote the conversion of HCC cells to the glycolytic pathway through upregulation of the different growth factors (insulin, IGF1) and ROS (179). Excessive adipose tissue expansion leads to reduced blood supply and induces a hypoxic environment, which in turn promotes adipose tissues and HCC cells glycolysis, leading to increased glucose consumption and lactate production. Thus, the excessive carbohydrate intake is mainly utilized by adipose tissue and HCC cells, sparing only a small portion for immune cells. This hypoxic, low-glucose, high-lactate environment inhibits liver-infiltrating immune effector cell function and proliferation, thus promoting the conversion of immune effector cells to immune suppressor cells. Obesity-induced inflammation not only facilitates oncogenesis via promoting a cycle of cell death-repair-fibrosis, but also leads to aging of immune effector cells such as T cells and B cells. Moreover, obesity also facilitates immune dysfunction via inducing lipid disorder. Cholesterol deposition in NK cells directly leads to their functional inhibition, thus producing less IFN-γ and expressing low levels of granzyme B as well as perforin (95). Increased FA uptake and FAO have been tightly related to enhanced immunosuppression in MDSC (99). Upregulated expression of CD36 as well as SREBP can result in augmented immunosuppressive function of Tregs, whereas lipid metabolism in TAMs was found to be involved in regulation of its immunosuppressive function (105, 108). The altered immune cell phenotype can directly lead to cytokine disruption, which in turn promotes tumor growth as well as immunosuppression.

Conventional therapy including surgery resection and systemic therapy is widely used in HCC treatment. The impact of obesity on HCC resection remains controversial. Many studies indicated that obesity has shown no effects on lethality rate after HCC resection, whereas several studies had shown that severe obesity increased lethality rate after HCC resection (181, 182). Besides, obesity appears to promote a range of complications after HCC resection (183, 184). Multi-kinase inhibitors (especially sorafenib) are widely used for the treatment of advanced HCC. The effect of obesity on sorafenib treatment has varied across clinical trials, which may be attributes to different obesity-related symptoms (185–187). In addition, body composition may affect the efficacy of sorafenib treatment. In brief, obesity upregulates overall survival rate after sorafenib when muscle mass is unaffected, whereas mortality increased when obesity leads to muscle loss, such as sarcopenia obesity (187–189). Considering that few studies have highlighted the effect of obesity on sorafenib efficacy during the past 16 years (since sorafenib was approved for HCC treatment in 2007), obesity may not be an important factor to predict responses to sorafenib. Recently, the emergency of immunotherapy suggests that people have better understanding of cancer progression. Although immunotherapy may be not significantly superior to sorafenib, it undoubtedly offers a new treatment strategy for HCC and provides an alternative treatment option when sorafenib tolerance occurs. Based on the fact that obesity can induce the onset of immunosuppression through affecting multiple pathways, the impact of obesity on immunotherapy appears to be paradoxical. Obesity predicts a better response to ICI regimens, perhaps because obesity can lead to increased levels of target marker proteins (which is also responsible for obesity-promoted HCC). Importantly, it remains unclear whether obesity is an independent factor that favors immunotherapy because most relative studies are clinical trials and mechanism studies are needed. Besides, enhanced responses to immunotherapy in obesity-related HCC may be attributed to relatively healthy state (when compared to patients with severe cancer-related disorders, such as cachexia, which is characterized by low BMI). In this circumstance, obesity may be just a marker of effective immunotherapy rather than independent factor. Since the immunosuppressive microenvironment caused by obesity undoubtedly hinders the function of immune effector cells, selective improvement of the obesity status according to application of the different immunotherapies (ICIs or adoptive cell therapy) might be a guaranteed therapeutic modality.

Above all, obesity can facilitate HCC progression via remodeling TIME and inducing immunosuppression, all of which mainly originate from the inflammatory environment but can exert pro-tumor effects once HCC has occurred. Given the tolerance to conventional therapy and the continued success of immunotherapy for HCC, more emphasis should be placed on obesity-induced immunosuppression. Investigations about obesity-related HCC progression from an immunosuppressive perspective can provide the basis for biomarker discovery and facilitate diagnostic evaluation as well as therapeutic development. However, it is unclear when the inflammatory environment might shift to immunosuppressive state, which may be an important direction for future insights into the development of obesity-related HCC.

## Author contributions

JY designed and drafted the manuscript. JH and YF revised the manuscript. MX designed and edited the manuscript. All authors contributed to the article and approved the submitted version.
